# Cerebellar Kainate Receptor-Mediated Facilitation of Glutamate Release Requires Ca^2+^-Calmodulin and PKA

**DOI:** 10.3389/fnmol.2018.00195

**Published:** 2018-06-06

**Authors:** Rafael Falcón-Moya, Pilar Losada-Ruiz, Talvinder S. Sihra, Antonio Rodríguez-Moreno

**Affiliations:** ^1^Laboratorio de Neurociencia Celular y Plasticidad, Departamento de Fisiología, Anatomía y Biología Celular, Universidad Pablo de Olavide, Sevilla, Spain; ^2^Department of Physiology, Pharmacology and Neuroscience, University College London, London, United Kingdom

**Keywords:** kainate receptors, glutamate, presynaptic, Ca^2+^-calmodulin, PKA, slices

## Abstract

We elucidated the mechanisms underlying the kainate receptor (KAR)-mediated facilitatory modulation of synaptic transmission in the cerebellum. In cerebellar slices, KA (3 μM) increased the amplitude of evoked excitatory postsynaptic currents (eEPSCs) at synapses between axon terminals of parallel fibers (PF) and Purkinje neurons. KA-mediated facilitation was antagonized by NBQX under condition where AMPA receptors were previously antagonized. Inhibition of protein kinase A (PKA) suppressed the effect of KA on glutamate release, which was also obviated by the prior stimulation of adenylyl cyclase (AC). KAR-mediated facilitation of synaptic transmission was prevented by blocking Ca^2+^ permeant KARs using philanthotoxin. Furthermore, depletion of intracellular Ca^2+^ stores by thapsigargin, or inhibition of Ca^2+^-induced Ca^2+^-release by ryanodine, abrogated the synaptic facilitation by KA. Thus, the KA-mediated modulation was conditional on extracellular Ca^2+^ entry through Ca^2+^-permeable KARs, as well as and mobilization of Ca^2+^ from intracellular stores. Finally, KAR-mediated facilitation was sensitive to calmodulin inhibitors, W-7 and calmidazolium, indicating that the increased cytosolic [Ca^2+^] sustaining KAR-mediated facilitation of synaptic transmission operates through a downstream Ca^2+^/calmodulin coupling. We conclude that, at cerebellar parallel fiber-Purkinje cell synapses, presynaptic KARs mediate glutamate release facilitation, and thereby enhance synaptic transmission through Ca^2+^-calmodulin dependent activation of adenylyl cyclase/cAMP/protein kinase A signaling.

## Introduction

Kainate-type glutamate receptors are well established mediators of canonical, ionotropic postsynaptic synaptic transmission and, presynaptically, the receptors support a modulatory regulation of neurotransmitter release. In the latter regard, kainate receptors (KARs) evince a non-canonical metabotropic capacity, through which they effect the control of both glutamate and GABA release (for review see Rodríguez-Moreno and Sihra, 2007a,b; Jane et al., 2009; Shira and Rodríguez-Moreno, 2013; Valbuena and Lerma, 2016). At several excitatory glutamatergic synapses, the KAR-mediated modulation is found to be biphasic, such that low agonist concentrations facilitate glutamate release, as opposed to higher agonist concentrations, which inhibit neurotransmitter release (for review see Rodríguez-Moreno and Sihra, 2007a,b, 2013; Lerma and Marques, 2013). How this diametrically opposite modulation is mechanistically manifest is subject of considerable debate and investigation, as is the question of the subcellular location of KARs responsible for presynaptic modulation (for review see Rodríguez-Moreno and Sihra, 2007a,b, 2013; Lerma and Marques, 2013).

KARs are expressed in the cerebellar cortex in the axons of cerebellar granule cells that form parallel fibers (PF), and make excitatory synapses with Purkinje cells (PuC, Smith et al., 1999). Messenger RNA transcripts encoding for different KAR subunits (GluK1, GluK2 and GluK5) have been detected in granule cells and functional expression of KAR subtypes has been reported (Bettler et al., 1990; Herb et al., 1992; Bahn et al., 1994; Petralia et al., 1994). The subunits GluK1 and GluK2 have been detected on parallel fibers, Petralia et al., 1994). Biophysical studies with single-channel recordings have shown GluK1 activity (Swanson et al., 1996), suggesting these KARs are Ca^2+^-permeable. A biphasic action of KARs, activated by the agonist domoate, has been shown previously at PF-PuC synapse, with low agonist concentrations facilitating synaptic transmission, and higher concentrations depressing synaptic transmission (Delaney and Jahr, 2002). However, the precise mechanism of action by which KARs mediate potentiation (and depression) of synaptic transmission at PF-PuC synapses is completely unknown. Here, we have examined the mechanism underpinning the facilitatory effect of KA in cerebellar slices at synapses between granule cell terminals and Purkinje cells.

First, establishing mechanistic features of the modulation, we found that the KAR-mediated facilitation of glutamate release and synaptic transmission has an obligatory dependency on adenylyl cyclase (AC) and cAMP-mediated protein kinase A (PKA) activity. Furthermore, the KAR-mediated modulation of transmission is reliant on both external Ca^2+^ entry via Ca^2+^-permeant KARs, and functional intracellular Ca^2+^-stores. Finally, obviation of facilitation by calmodulin inhibition invokes a mechanistic coupling of KARs through Ca^2+^-calmodulin/AC/cAMP/PKA signaling, at PF-PuC synapses in the cerebellum.

## Materials and Methods

### Animals

The experiments were performed on 4–6 week old C57Bl/6 male mice obtained from Harlan Laboratories (Spain). Experiments were conducted in accordance with the European Union directive for the use of laboratory animals in acute experiments and were approved by the local Ethical Committee (Junta de Andalucía and University Pablo de Olavide, Sevilla, Spain).

### Slice Preparation

Cerebellar parasagittal slices were prepared. Briefly, after decapitation, the whole brain was removed under ice-cold buffered salt solution consisting of (in mM) 124 NaCl, 2.69 KCl, 1.25 KH_2_PO_4_, 2 MgSO_4_, 1.8 CaCl_2_, 26 NaHCO_3_, and 10 glucose (pH 7.2, 300 mOsm), and positioned on the stage of a vibratome slicer (Leica 1000S), and cut to obtain cerebellar slices (350 μm thick) containing parallel fibers-Purkinje cells synapses. Slices were maintained continuously oxygenated for at least 1 h before use. All experiments were carried out at room temperature (22–25°C). During experiments, slices were continuously perfused with buffered salt solution as detailed above.

### Electrophysiological Recordings

Whole-cell patch-clamp recordings were made from Purkinje neurons. NMDA receptor-mediated evoked excitatory postsynaptic currents (eEPSCs) were recorded at +40 mV from these neurons visually identified by infrared-differential interference contrast (IR-DIC) microscopy using a 40× water immersion objective. Perfusion solution contained GYKI53655 (30 μM), to block AMPA receptors, and bicuculline (10 μM), to block GABA_A_ receptors. In experiments involving AMPA receptor-mediated currents, performed at −70 mV, no GYKI53655 was used, but D-AP5 (50 μM) was included to block NMDA receptors. To evoke eEPSCs, electrical pulses were delivered to granule cells axons (parallel fibers) using a monopolar electrode placed in the molecular layer at a frequency of 0.2 Hz. Patch electrodes were made from borosilicate glass and had a resistance of 4–7 MΩ when filled with (mM): 120 CsCl, 8 NaCl, 1 MgCl_2_, 0.2 CaCl_2_, 10 HEPES, 2 EGTA and 20 QX-314 (pH 7.2, 290 mOsm). A 40 ms paired-pulse stimulation protocol was used for pair pulse ratio (PPR) analysis. Neurons were voltage clamped, using a Multiclamp 700B amplifier (Molecular Devices, Foster City, CA, USA). Access resistance was regularly monitored during recordings, and cells were rejected if it changed >15% during the experiment. Data were filtered at 2 kHz, digitized at 10 kHz, and stored on a computer using pClamp software (Molecular Devices). Synaptic failures were identified as the lack of synaptic responses after presynaptic stimulation with the amplitude of these responses being no different from basal noise amplitude.

### Data Analysis

Data were normalized taking the control as 100% of the response and presented as means ± SEM. Signals were averaged every 12 traces. Effects of KA were measured at peak (maximum) compared to averaged 10 min baseline points. Significance was assessed at *P* < 0.05. Statistical comparisons were made using two-tailed Student’s *t*-test for comparison of two data sets and ANOVA for comparison of multiple data set using the Bonferroni as a *post hoc* test.

### Compounds

Salts and general reagents were purchased from Sigma (St. Louis, MO, USA); GYKI 53655, D-AP5, NBQX, bicuculline, Rp-Br-cAMP, H-89, forskolin, philanthotoxin, ryanodine, thapsigargin, kainate, Pertussis toxin CMZ and W-7 were obtained from Tocris (Bristol, UK).

## Results

### The Activation of Kainate Receptors By 3 μM KA Produces an Increase in the Amplitude of NMDA-Evoked Postsynaptic Currents at PF-PuC Synapses

Following the observation that glutamatergic transmission at PF-PuC synapses of juvenile rats pups is modulated by KARs in a biphasic manner (Delaney and Jahr, 2002), as is also the case in the hippocampus (for review see Rodríguez-Moreno and Sihra, 2007a,b, 2013; Lerma and Marques, 2013), we established the parallel fiber-Purkinje (PF-PuC) synapse paradigm in slices from early adult mouse cerebellum.

**Figure 1:**
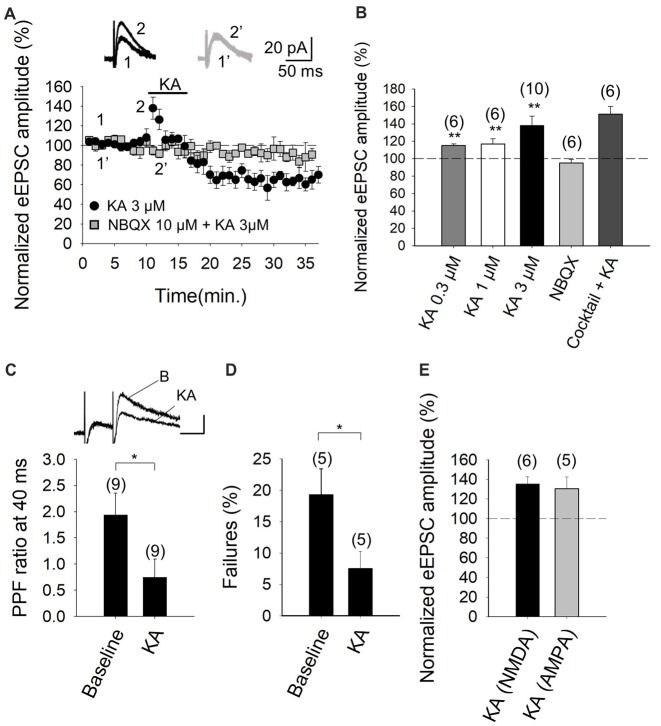
Kainate (KA) increases the evoked excitatory postsynaptic currents (eEPSCs) amplitude at parallel fibers-Purkinje cells (PF-PuC) synapses of the cerebellum. **(A)** Time course of KA (3 μM) effect on eEPSCs amplitude in the absence (circles) and presence of NBQX (squares). Inset show traces before and after 4 min KA perfusion in the absence (1, 2) and in the presence of 10 μM NBQX (1′, 2′). **(B)** Quantification of modulation observed in **(A)** and dose dependency. **(C)** KA (3 μM) perfusion produces a decrease of the paired pulse ratio, inset shows scaled representative traces. **(D)** Effect of KA on the number of failures of NMDA receptor-mediated currents. **(E)** Effect of KA (3 μM) on NMDA and AMPA receptor-mediated currents, respectively. Note that the effect of kainate on these currents is indistinguishable. The number of slices (from two to three mice) is indicated in parenthesis at the top of each bar. Results are expressed as means ± SEM (*P < 0.05, **P < 0.01, Student’s *t*-test).

The experimental paradigm we used was the stimulation of parallel fiber axons while measuring NMDA receptor-mediated eEPSCs in PuCs, by whole-cell patch clamp recordings, with the membrane potential held at +40 mV. Recording were made in the presence of 30 μM GYKI53655, in order to obviate AMPA receptor activation, as well as the presence of 10 μM bicuculline, to antagonize GABA_A_ receptors. In our experiments, young adult cerebellar synapses evince detectable facilitation of NMDA receptor-mediated eEPSC amplitudes at 3 μM KA (138 ± 11%, *n* = 10, Figures 1A,B), with 0.3 μM and 1 μM agonist concentrations having smaller effects (115 ± 2%, *n* = 6, 117 ± 6%, *n* = 6, respectively). With 3 μM KA, synaptic facilitation was followed by a 36 ± 8% (to 64 ± 8% of baseline, *n* = 10) decrease in the eEPSC amplitude (Figure 1B). To analyze the mechanistic details of the KAR-mediated facilitation of glutamatergic transmission, we hereafter utilized 3 μM KA in subsequent electrophysiological experiments as 3 μM KA produced the maximum level of facilitation observable (Figures 1A,B).

To determine whether the effect of KA recorded from Purkinje neurons in slices was mediated by the activation of KARs, analogous to that observed in other brain regions, such as the hippocampus and cortex (Lauri et al., 2001a,b, 2003; Schmitz et al., 2001; Ji and Stäubli, 2002; Contractor et al., 2003; Breustedt and Schmitz, 2004; Rodríguez-Moreno and Sihra, 2004, 2013; Campbell et al., 2007; Pinheiro et al., 2007; Scott et al., 2008; Fernandes et al., 2009; Jouhanneau et al., 2011; Andrade-Talavera et al., 2012, 2013), we performed experiments in the presence of NBQX. We showed that the 3 μM KA biphasic effect on the eEPSC amplitude was abolished in the presence of 10 μM NBQX (95 ± 4%, *n* = 6, Figures 1A,B). In these experiments, because AMPA receptors are antagonized in the presence of the selective blocker GYKI53655 in the bath, the observation of full antagonism by NBQX invokes the modulation to be due to KARs specifically. Further, in line with the notion that the facilitation (and the depression) of synaptic transmission observed is exclusively contingent on KAR activation. KA-mediated facilitation was retained (151 ± 9% increase of eEPSCs amplitude, *n* = 6) when other transmitter influences were obviated by the inclusion of a cocktail of inhibitors including the receptor antagonists: MCPG and MPPG (1.5 mM), naloxone (100 μM), bicuculline (20 μM), 2-OH-saclofen (150 μM), atropine sulfate (50 μM) and DPCPX (0.1 μM), to block metabotropic glutamate, opioid, GABA_A_, GABA_B_, muscarinic and adenosine receptors, respectively. Indeed, the synaptic depression that followed the facilitation of EPSCs, was also present in the presence of the inhibitor cocktail (to 65 ± 8% of the baseline, *n* = 6). These data therefore exclude the possibility that KA-mediated modulation was a secondary consequence of the synaptic release of diverse neurotransmitters, but rather, support the hypothesis that a direct effect of KA on KARs at cerebellar synapses underpins the observed modulation.

Previously, the facilitatory action of KA at the PF-PuC synapses have been attributed to presynaptic regulation (Delaney and Jahr, 2002). In our studies, we confirmed a presynaptic locus of action by using several approaches. First, we performed paired-pulse recordings and measured the paired-pulse ratio (PPR; pair-pulse depression was observed at 40 ms pulse interval). PPR was 1.9 ± 0.4 (*n* = 9) under control/baseline conditions. After KA treatment, PPR decreased to 0.7 ± 0.3 (*n* = 9; Figure 1C), implying an effect on release probability (Manabe et al., 1993), thereby corroborating the presynaptic origin of the KA receptor-mediated regulation. Second, we determined the proportion of synaptic failures in presence of KA. Under control conditions, synaptic failure rate was 19 ± 4%, *n* = 5. Following the application of KA, the failure rate was measurably decreased (to 7 ± 3%, *n* = 5, Figure 1D), again indicating a presynaptic locus of KA action. Finally, we compared the KA-mediated modulation of NMDA receptor-mediated eEPSCs (with GYKI53655 present) and AMPA receptor-mediated eEPSCs recorded at −70 mV (without GYKI53655, but with D-AP5 and bicuculline, to respectively block NMDA and GABA_A_ receptors). KA mediated a comparable increase in the NMDA receptor-mediated eEPSCs (135 ± 7%, *n* = 6, Figure 1E) and the AMPA receptor-mediated eEPSCs (130 ± 12%, *n* = 5, Figure 1E). This congruent facilitation of NMDA and AMPA receptor-mediated eEPSC amplitudes intimates that KA-modulation acts upstream of postsynaptic receptor activation, i.e., at the level of presynaptic terminal, through increased glutamate release. Together the preceding evaluation reliably evinces a presynaptic locus of action of KA at the PF-PuC synapses under investigation. However, it remains to be seen where the respondent KARs are physically located i.e., terminal, axonal or somatodendritic presynaptic compartments.

### KAR-Mediated Facilitation of Glutamatergic Transmission at PF-PuC Is Contingent on cAMP-Dependent Signaling

**Figure 2:**
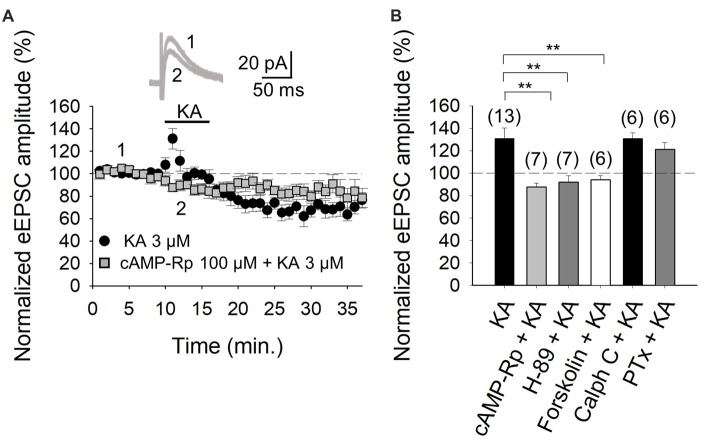
Activation of adenylyl cyclase (AC) and downstream protein kinase A (PKA) underlies the kainate-mediated facilitation of glutamate release in PF-PuC synapses. **(A)** Time-course of the effect of KA on eEPSC amplitude in cAMP-Rp treated slices. Inset shows representative traces showing that KA (3 μM) does not increase the amplitude of the eEPSCs in cAMP-Rp treated slices. **(B)** Inhibition of PKA by cAMP-Rp (100 μM) or H-89 (2 μM) and activation of AC by forskolin (30 μM) prevented the facilitatory action of KA. Inhibition of protein kinase c (PKC) with calphostin C (1 μM) has no effect on the KA enhancement of the eEPSC amplitude. The facilitatory effect of KA is not affected in slices treated with pertussis toxin. The number of slices (from two to three mice) is indicated in parenthesis at the top of each bar. Results are expressed as means ± SEM (**P < 0.01, ANOVA test).

With the selectivity of the action of KA verified, in subsequent experiments we examined the second messenger system that mediates the facilitation of eEPSCs. First, we tested whether PKA was involved in the increased eEPSCs, by inhibiting either the cAMP-mediated activation of PKA, or the catalytic activity of the kinase, by respectively treating slices with the inhibitors cAMP-Rp or H-89. With the addition of 100 μM cAMP-Rp or 2 μM H-89, the facilitation of the eEPSC amplitude by 3 μM KA was eliminated (88 ± 3%, *n* = 7 after cAMP-Rp and 92 ± 6%, *n* = 7 after H-89, vs. KA 3 μM, 131 ± 9%, *n* = 13 Figures 2A,B). cAMP-Rp and H-89 alone produced only small decrements in the eEPSC amplitude (7 ± 3% and 10 ± 4%, respectively, *n* = 5, data not shown). The data together point to PKA playing an obligatory part in the observed KA-mediated modulation of PF-PuC cerebellar glutamatergic transmission.

For further corroboration, we next examined the KA-mediated facilitation of eEPSCs in presence of direct AC activation by forskolin. Given that forskolin produces long-lasting effects (Tong et al., 1996), slices were preincubated for 1 h with the diterpene in these experiments. KA application to forskolin (30 μM)-treated slices failed to alter eEPSC amplitudes (95 ± 4%, *n* = 6, Figure 2B). This observation evinces that the previous AC activation by forskolin, obviates KAR-mediated regulation at PC-PuC synapses. To ensure that the forskolin-mediate abrogation of KAR regulation was indeed due the elevation of cAMP production, rather than a non-specific effect, we used 1,9-dideoxyforskolin, the inactive diterpine analog of forskolin, in control experiments. With dideoxyforskolin (100 μM) incubation of slices, KA (3 μM) invoked a facilitation of 42 ± 5% (to 142 ± 5% of baseline, *n* = 5) in the amplitude of the eEPSCs, comparable to KA controls. This corroborates that occlusion of the KA receptor-mediated modulation by forskolin can indeed be ascribed to an increase in cAMP levels due to a pharmacological stimulation of AC. In sum, these data support the hypothesis that forskolin, through an AC/cAMP/PKA pathway, occludes the metabotropic, facilitatory and presynaptic action of KARs at PF-PuC synapses.

The aforementioned data in Figure 2 indicate that the KA receptor-mediated facilitation of glutamatergic transmission at these cerebellar PF-PuC synapses is manifest through an AC/cAMP/PKA signaling pathway. However, given that in other slice preparations, protein kinase C (PKC) has also been implicated in aspects of the KAR-mediated modulation (for reviews see Rodríguez-Moreno and Sihra, 2007a,b), we examined whether this kinase plays a role in the modulation of the PF-PuC cerebellar synapse by KA. In slice experiments using calphostin C (1 μM) to specifically inhibit PKC, no significant effect on the KA-mediated facilitation was observed (131 ± 5%, *n* = 6, Figure 2B), this result therefore obviating involvement of PKC in the modulation observed.

Next, we determined whether the facilitatory effect of presynaptic KAR activation involves G-protein function, by examining the effect of KA on slices treated with Pertussis toxin (PTx, 5 μg/ml). Intriguingly, in the presence of PTx, the KAR-mediated facilitation of synaptic transmission was retained, unaffected by G-protein block (122 ± 6%, *n* = 6, vs. 131 ± 9, *n* = 13 without PTx, Figure 2B). Interestingly, and as an important positive control for the activity of PTx, we found that the inhibitory effect of KA at the same synapse was indeed suppressed by PTx; implying selective G-protein involvement in inhibitory modulation by KAR in the preparation under study (90 ± 8%, *n* = 6; vs. control 65 ± 7%, *n* = 13, not shown).

### Facilitation of Synaptic Transmission/Glutamate Release at PF-PuC Synapses Is Mediated by a Ca^2+^ Permeant Presynaptic KAR: Contingency on the Mobilization of Intracellular Ca^2+^ Stores and Ca^2+^/Calmodulin Dependence

**Figure 3:**
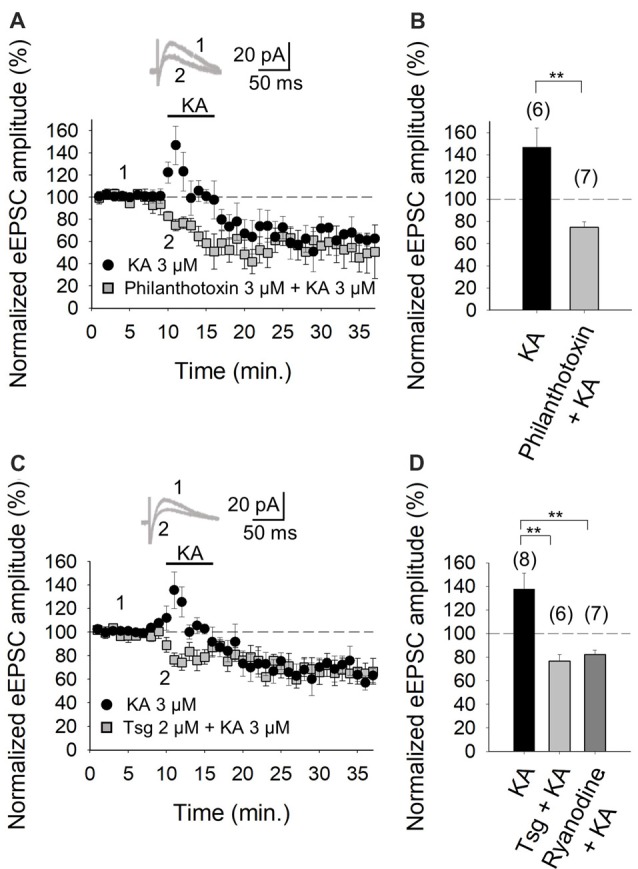
Facilitation of glutamate release mediated by presynaptic kainate receptor (KAR) activation requires an increase of Ca^2+^ in the cytosol at PF-PuC synapses. **(A)** Time-course of KA (3 μM) effect on eEPSCs amplitude in control condition (circles) and in slices treated with philanthotoxin (squares). **(B)** Quantification of modulation observed in **(A)**. **(C)** Time-course of the effect of KA on eEPSCs amplitude in control slices (circles) and in thapsigargin-treated slices (squares). **(D)** In slices treated with thapsigargin or ryanodine, the increase of eEPSCs amplitude induced by KA is prevented. The number of slices (from two to three mice) is indicated in parenthesis at the top of each bar. Results are expressed as means ± SEM (**P < 0.01, Student’s *t*-test and ANOVA).

The role of Ca^2+^ in mediating KAR-mediated synaptic facilitation has been subject of debate and controversy, for instance, at the hippocampal mossy fiber-CA3 (MF-CA3) synapses. Some studies suggest that permeation of Ca^2+^ through KARs and subsequent Ca^2+^-induced Ca^2+^ release from intracellular stores is obligatory for short-term and long-term plasticity at MF-CA3 synapses (Lauri et al., 2003; Scott et al., 2008). Others have registered no effect of KA on cytosolic [Ca^2+^] (Kamiya et al., 2002) and yet others advocate that a decrement Ca^2+^ concentration underpins the modulation due to KAR activation (Kamiya and Ozawa, 1998, 2000). To examine the former possibility and the presence of Ca^2+^ permeant KARs at these cerebellar synapses, we investigated the effect of KA on the eEPSC amplitudes in the presence of philanthotoxin, a toxin shown to block unedited, Ca^2+^ permeable KARs (Fletcher and Lodge, 1996; Scott et al., 2008). After treatment of slices with 3 μM philanthotoxin, the synaptic facilitation mediated by 3 μM KA was completely abrogated (to 75 ± 5% of initial amplitude, *n* = 7 vs. 147 ± 17%, *n* = 6 observed in interleaved slices, Figures 3A,B). These results evince that Ca^2+^ permeation through KAR is essential for the synaptic facilitation observed at PF-PuC synapses. To establish whether the aforementioned Ca^2+^ signal produced by KAR activation required amplification by Ca^2+^-induced intracellular Ca^2+^-store mobilization, we examined the effect of KA following depletion of intracellular Ca^2+^-stores, using thapsigargin to inhibit the SERCA pump responsible for Ca^2+^ accumulation into the stores. Treatment with thapsigargin (2 μM) eliminated the facilitatory effect of 3 μM KA, and, indeed, rather produced a depression of the response (77 ± 5%, *n* = 6, with thapsigargin vs. 138 ± 13%, *n* = 8, without thapsigargin, in interleaved slices; Figures 3C,D). These data unequivocally demonstrate the mandatory requirement for intracellular Ca^2+^ stores in the modulation elicited by KA. However, the question remains whether KA treatment mobilizes these intracellular Ca^2+^ stores by first triggered an initial rise of cytosolic Ca^2+^ concentrations due to extracellular Ca^2+^ influx. To answer this, we examined the effect of ryanodine, which selectively inhibits Ca^2+^-induced Ca^2+^ release (Berridge, 1998), to elucidate whether this underpins KAR-mediated facilitation. Ryanodine (10 μM) treatment eliminated the KAR-mediated facilitation of transmission at these PF-PuC synapses (82 ± 4%, *n* = 7, with ryanodine vs. 138 ± 13%, *n* = 7, without ryanodine, in interleaved slices; Figure 3D). Taken together, the foregoing results support the hypothesis that presynaptic KARs at PF-PuC synapses are Ca^2+^ permeable, and that Ca^2+^ entry effected by these KARs and Ca^2+^ channels, triggers Ca^2+^-induced Ca^2+^-release from intraterminal Ca^2+^-stores to produce synaptic facilitation.

**Figure 4:**
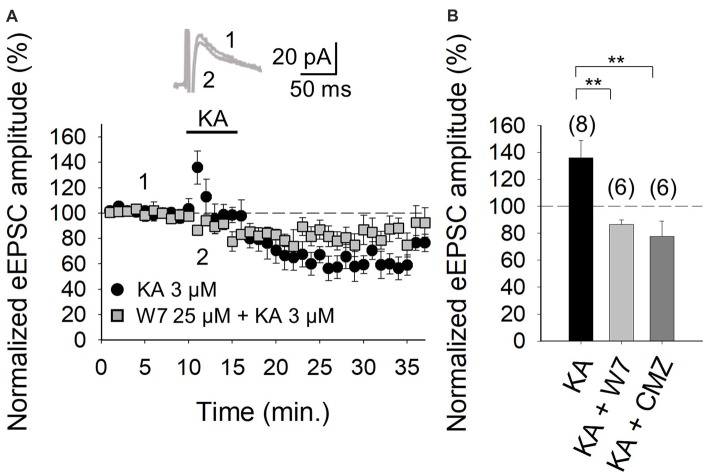
Facilitation of glutamate release mediated by presynaptic KAR activation requires Ca^2+^-calmodulin at PF-PuC synapses. **(A)** Time-course of KA (3 μM) effect on eEPSCs amplitude in control condition (circles) and in the slices treated with 25 μM W-7 (squares). Inset show traces before and after 4 min KA perfusion from W-7 treated slices. **(B)** Quantification of modulation observed in **(A)** and in the presence of 1 μM CMZ. The number of slices (from two to three mice) is indicated in parenthesis at the top of each bar. Results are expressed as means ± SEM (**P < 0.01, ANOVA test).

It is evident from the foregoing data that an entry of Ca^2+^ via KARs is obligatory for the mediation of the facilitation invoked by KA. Given that in the hippocampus and the cortex, where we have previously examined KAR-mediated modulation, the activation of a Ca^2+^-calmodulin complex was shown to be mandatory for AC activation (Andrade-Talavera et al., 2012, 2013), we examined this regulatory pathway at the cerebellar PF-PuC synapse by treating slices with the calmodulin antagonist, W-7, before recording eEPSCs. With W-7 (25 μM) present, KA (3 μM)-mediated facilitation was convincingly blocked (86 ± 3%, *n* = 6, with W-7 vs. 136 ± 13%, *n* = 8 without W7, in interleaved slices; Figures 4A,B). Corroboration of calmodulin dependence of the modulation, was also evident from experiments performed in the presence of calmidazolium (CMZ, 1 μM), an alternative calmodulin antagonist. As with W-7, in presence of CMZ, KA (3 μM)-mediated facilitation of synaptic transmission was abograted (78 ± 11, *n* = 6, Figure 4B). These data support the postulate that a presynaptic Ca^2+^-calmodulin complex is obligatory for the KAR-mediated synaptic regulation and operates upstream of the activation of AC/cAMP/PKA signaling.

## Discussion

The results in this study, utilizing electrophysiological experiments in cerebellar slices, show that the activation of presynaptic KARs in the cerebellum, at PF-PuC synapses, invokes a facilitation of synaptic transmission/glutamate release. Analysis of this modulation suggests a mechanistic coupling of KARs through Ca^2+^-calmodulin/AC/cAMP/PKA activity, but independently of G-protein activation.

We would hypothesize that the observed KA-mediated enhancement of the eEPSPs at PF-PuC synapses is due to increased glutamate release, which could be monitored by NMDA receptor-mediated currents (with AMPA receptors antagonized by GYKI53655), and blocked by the KAR/AMPA receptor antagonist NBQX. Under conditions where AMPA receptor activation is obviated by GYKI53655, this therefore delineates the specific role of KARs in the facilitatory regulation, particularly given that a receptor antagonist cocktail, formulated to eliminate the presynaptic effects of neurotransmitters that might be secondarily released, also had no effect on the observed synaptic modulation due to KA.

In assessing synaptic regulation, it is indeed of utmost importance to identify the subcellular location of the KAR postulated. We corroborated the presynaptic presence of KARs by electrophysiological analysis of a presynaptically manifest parameter, i.e., the PPR (pair-pulse ratio) of consecutive eEPSCs mediated by neurotransmitter release. A clear decrease in the PPR of eEPSCs observed with KA application in our experiments, suggested a change in release probability (by definition a presynaptic property in synaptic transmission). Secondly, we assessed the proportion of synaptic failures in response to KA application. With KA application, the failure proportion was evidently diminished, supportive of an increase in the probability of presynaptic transmitter release and corroborative of the observed facilitation occurring through KAR activation. Finally, importantly, the effects of KA observed on NMDA and AMPA receptor-mediated currents were similar. Given that no such equivalence would be predicted if the observed modulation was postsynaptic, the data here are supportive of a presynaptic mode of action for KARs being activated. Altogether, three independent analyses mutually corroborate and emphasize a presynaptic locus of action of KARs functioning at PF-PuC synapses.

It remains to be elucidated whether the presynaptic regulation by KA at PF-PuCs reports the activity of KARs subcellularly localized at nerve terminal/axonal or somatodendritic compartments. The technically challenging paradigms needed to address this question is beyond the scope of the present paper. However, to directly elucidate the presynaptic compartmentalization of KARs, future work necessitates: (i) high resolution immunolocalization (immunogold-based) of the receptor (contingent on the availability of high affinity antibodies with appropriate KAR subunit-specificity); and (ii) targeted blockade of KARs using caged-antagonists (contingent on the pending development of reagents) (see NMDA receptor studies, Rodríguez-Moreno et al., 2011; Reeve et al., 2012).

Corresponding with our previous studies in the hippocampus and cortex (Rodríguez-Moreno and Sihra, 2004, 2013; Andrade-Talavera et al., 2012, 2013), PKA inhibition by the cell-permeant cyclic nucleotide analog cAMP-Rp, also led to the abrogation of KA-mediated enhancement of synaptic transmission/glutamate release at PF-PuC synapses. The congruence of the mechanism between synapses was also highlighted in the current studies, by the observation that the inhibition PKA catalytic activity by H-89 also eliminated the KA-mediated facilitation. Similarly and congruently, direct activation of AC by preincubation with forskolin, produced refractoriness of the facilitatory effect of KA. Collectively, these results consistently suggest that AC/cAMP/PKA signaling underpins the facilitatory modulation of synaptic transmission/glutamate release at the synapse under study here.

Consistently, there has been a notable absence of any evidence to support G-protein mediated initiation/transduction of the AC/cAMP/PKA cascade posited to be involved in presynaptic KAR-mediated enhancement of glutamate release. We therefore addressed the conceivable role of Ca^2+^ as the initiator of a G-protein independent signaling cascade, in the modulation being investigated at defined cerebellar PF-PuC synapses. In the canonical context, KARs may mediate external Ca^2+^ entry through an ionotropic activity which would depolarize nerve terminals (Perkinton and Sihra, 1999) and thus activate voltage-gated Ca^2+^ channels. Non-canonically, direct Ca^2+^ influx via Ca^2+^ permeable KARs *per se* is also possible (Fletcher and Lodge, 1996; Scott et al., 2008). Notably in the studies reported herein, a blockade of the Ca^2+^ permeable KARs by the selective inhibitor, philanthotoxin, abrogated the KA-mediated synaptic facilitation in the current studies, pointing to a strict requirement for external Ca^2+^ entering via KARs to support facilitatory modulation. Interestingly therefore, it would appear that, although it is thought that unedited Ca^2+^ permeable KARs typically prevail earlier during neuronal development, these receptors evidently persevere with activity in the cerebellum of the early adult, mouse brain.

We extended the analysis of properties of KAR-mediated regulation by examining the hypothesis that the essential core, albeit perhaps limited, Ca^2+^ entry via KARs may be amplified by Ca^2+^ mobilized from intraterminal stores, as reported for similar modulation at hippocampal synapses (Lauri et al., 2003; Scott et al., 2008). Emphatically supporting a critical role for intraterminal Ca^2+^ stores, were our results showing that thapsigargin treatment, to deplete intracellular Ca^2+^ stores (Irving et al., 1992), abolished the facilitatory regulation by KARs. Again, in corroboration of the hypothesis, use of ryanodine to selectively inhibit Ca^2+^-induced Ca^2+^-release (Berridge, 1998), evinced that Ca^2+^ entry via KARs induces Ca^2+^ mobilization from intraterminal Ca^2+^ stores to invoke the modulation seen herein.

Having demonstrated that Ca^2+^ entering via KARs and subsequence Ca^2+^ increases due to mobilization of intraterminal Ca^2+^ stores is actually obligatory for the KA facilitation of synaptic transmission at PF-PuC synapse, we questioned how might such an increase in cytosolic [Ca^2+^] couple to the postulated AC/cAMP/PKA signaling mediating the facilitation by KARs. From previous studies, it is plausible that the rise in cytosolic [Ca^2+^] activates Ca^2+^-dependent ACs present in parallel fiber terminals. A number of ACs have been described, however, AC1 and AC8 are two members of the family, that have been shown to be activated by Ca^2+^-calmodulin and are abundant in the central nervous system (for reviews see Cooper, 2003; Wang and Storm, 2003). Interestingly, supporting their significance to the hypothesis, studies with double knockouts of AC1 and AC8, have shown that the Ca^2+^-calmodulin ACs are indeed essential for the widely reported Ca^2+^-dependent elevation of cAMP (Wong et al., 1999). Using the calmodulin antagonists W-7 and CMZ, our data shows that inhibiting Ca^2+^-calmodulin function, abolishes the presynaptic KAR-mediated modulation in cerebellar slices. This supports the hypothesis that, following KAR activation and elevation of cytosolic [Ca^2+^], a Ca^2+^-calmodulin-dependent coupling may activate AC1 and/or AC8, and thereby extend to the initiation of the AC/cAMP/PKA cascade, hence promoting synaptic facilitation through increased neurotransmitter release at PF-PuCs synapses.

This report shows the PF-PuC synapse is a reliable and robust model for the study of KAR mediated modulation. Interestingly, however, we observed a facilitation of synaptic transmission/glutamate release at 3 μM KA, contrary to previous work with the same synapse, where 500 nM agonist induced a depression in glutamate release (Delaney and Jahr, 2002). The discrepancy may reflect the notably different agonist concentrations employed, and indeed the difference in the age of animals, and perhaps the species of animals used in the previous experiments (Delaney and Jahr, 2002 utilized P13-P17 rats). The higher concentration of KA necessary for the activation of KARs at young adult synapses may indeed be indicative of the different efficacy of KA at KARs at this age, perhaps dependent on the expression of specific glutamate receptor subunit subtypes composing the resident KARs.

From our results, it is clear that KAR function is preserved in early adult animals at PF-PuC synapses and is not temporally limited to the two firsts postnatal weeks as reported previously (Delaney and Jahr, 2002). KARs have an autoreceptor role in developing animals, with the concentration of agonist determining presynaptic modulation: facilitation (at low [KA]) and depression (at high [KA]) and, thereby, putatively determining synapse consolidation and stability. Although the precise role(s) of these KARs in adult animals remains to be explicated, the modulation of presynaptic function reported herein might manifest some forms of plasticity. For instance, KARs have been shown to be involved in plasticity at PF-PuCs synapses (for review see Hirano, 2013; Sihra et al., 2014).

Our experiments here do show that KAR activation has a biphasic effect at PF-PuC synapses as previously reported (Delaney and Jahr, 2002); inducing a depression of glutamate release at relatively high concentrations of KA (>1–3 μM) rather than facilitation. The aim of the current study was to elucidate the mechanistic details of the observed facilitation, rather than the factors underpinning in the synaptic depression. However, notably, our results did reveal that the transient synaptic depression observed with high KA concentrations is abolished in the presence of cAMP-Rp (inhibition of PKA activation), but not affected by any of the experimental manipulations speaking to regulation of cytosolic [Ca^2+^] or function thereof, i.e., philantotoxin, thapsigargin, W-7 or CMZ. These observations point to the synaptic depression seen likely involving an AC/cAMP/PKA pathway, as described for facilitation, but without the proposed Ca^2+^-AC coupling. The indications are therefore, that KARs have alternative mechanistic modes for facilitatory and depressive action. Indeed, KAR coupling to an increase in cAMP concentrations (and subsequent enhancement of PKA activity), or to a decrease in cAMP concentrations (and subsequent diminution of PKA activity), has been reported in studies investigating the mossy fiber-CA3 synapse of the hippocampus (Negrete-Díaz et al., 2006, 2007; Andrade-Talavera et al., 2012), the amygdala (Negrete-Díaz et al., 2012) and in the cortex (Andrade-Talavera et al., 2013). The key difference that our current results point to is the differing direction and means of regulation of the AC/cAMP/PKA cascade in the bimodal regulation by KARs. Whereas, presynaptic facilitatory function by KARs evidently involves an increase in AC/cAMP/PKA signaling instigated by the Ca^2+^-calmodulin complex, KARs appear to be negatively coupled to the AC/cAMP/PKA pathway to effect depression synaptic transmission. Previous studies at MF-CA3 synapses and thalamocortical synapses (and as confirmed here) have reported that the depression of presynaptic function occurs through a negative coupling to AC/cAMP/PKA and is actually invoked by the action of a PTx sensitive G-protein (Negrete-Díaz et al., 2006; Andrade-Talavera et al., 2013). Notwithstanding the postulated differential upstream coupling to AC to achieve facilitation and depression, it is also plausible that the diametric mechanisms reflect the operation of two distinct types of KARs. Future studies will elucidate the exact instruments involved in the observed KAR-mediated modulation and address the question as to whether different populations of presynaptic KARs reside at the PF-PuC synapse.

In conclusion, our studies show that presynaptic KARs activation by KA, at PF-PuC synapses produces a facilitation of synaptic transmission consistent with an increase in neurotransmitter release. We postulate that, mechanistically, KAR-mediated presynaptic facilitation involves an increase in cytosolic [Ca^2+^], first by external Ca^2+^-entry via Ca^2+^-permeable KARs, and second by this then triggering the mobilization of intracellular Ca^2+^ from stores in granule cells terminals. The raised Ca^2+^ binds to calmodulin to form a Ca^2+^-calmodulin complex, which we postulate activates AC1 or AC8 to elevate cAMP levels and thus effect PKA stimulation. The latter invokes an enhancement of glutamate release and hence synaptic transmission at the PF-PuC synapse in the cerebellum.

## Author Contributions

RF-M and PL-R performed the experiments. TS and AR-M wrote the manuscript. AR-M designed the study.

## Conflict of Interest Statement

The authors declare that the research was conducted in the absence of any commercial or financial relationships that could be construed as a potential conflict of interest.
